# Supplementary material for: Household air pollution and risk of pulmonary tuberculosis in HIV-Infected adults
Source: Environ Health. 2024 Jan 17;23:6. doi: 10.1186/s12940-023-01044-0 (PMC10792790; doi:10.1186/s12940-023-01044-0)
Supplement: Supplementary file 1 — Additional file 1: eFigure 1. Twenty-four-hour (h) time-weighted average (TWA) and maximum (MAX) personal CO concentrations among 96 cases (HIV+/TB+) and 159 controls (HIV+/TB-). Horizontal lines indicate medians and interquartile ranges. eFigure 2. Twenty-four-hour (h)-weighted average (TWA) and maximum (MAX) personal CO concentration among 196 women and 58 men HIV-infected patients in South-Kivu, DR Congo. Horizontal lines indicate medians and interquartile ranges. eTable 1. Time-weighted average concentrations (ppm) of carbon monoxide among 255 HIV-infected outpatients attending ART-clinics and among their neighbours (n=97) in South Kivu, DRC. eTable 2. Multivariable Analysis: Quintiles of 24h Personal CO as Predictors for Tuberculosis Among 255 HIV-Infected Outpatients Attending ART-Clinics in South Kivu, DRC. [file 12940_2023_1044_MOESM1_ESM.docx]

**Additional file 1: eFigure 1.** Twenty-four-hour (h) time-weighted average (TWA) and maximum (MAX) personal CO concentrations among 96 cases (HIV+/TB+) and 159 controls (HIV+/TB-). Horizontal lines indicate medians and interquartile ranges. **eFigure 2.** Twenty-four-hour (h)-weighted average (TWA) and maximum (MAX) personal CO concentration among 196 women and 58 men HIV-infected patients in South-Kivu, DR Congo. Horizontal lines indicate medians and interquartile ranges. **eTable 1.** Time-weighted average concentrations (ppm) of carbon monoxide among 255 HIV-infected outpatients attending ART-clinics and among their neighbours (n=97) in South Kivu, DRC. **eTable 2.** Multivariable Analysis: Quintiles of 24h Personal CO as Predictors for Tuberculosis Among 255 HIV-Infected Outpatients Attending ART-Clinics in South Kivu, DRC
